# A Network Pharmacology Approach to Uncover the Molecular Mechanisms of Herbal Formula Kang-Bai-Ling for Treatment of Vitiligo

**DOI:** 10.1155/2019/3053458

**Published:** 2019-11-03

**Authors:** Manyuan Xu, Jianxin Shi, Zhongsheng Min, Hongliu Zhu, Weiguo Sun

**Affiliations:** ^1^Department of Dermatology, The Affiliated Huaian No. 1 People's Hospital of Nanjing Medical University, Huaian 223300, China; ^2^Department of Dermatology, Jiangsu Province Hospital of Chinese Medicine, Nanjing 210029, China; ^3^Department of Dermatology, Jiangyin Hospital of Traditional Chinese Medicine, Jiangyin 214400, China

## Abstract

**Background:**

Kang-bai-ling (KBL), a Chinese patent medicine, has been demonstrated as an effective therapy for vitiligo in China. However, the pharmacological mechanisms of KBL have not been completely elucidated.

**Methods:**

In this study, the potential multicomponent, multitarget, and multipathway mechanism of KBL against vitiligo was clarified by using network pharmacology-based strategy. In brief, potential targets of KBL were collected based on TCMSP databases, followed by network establishment concerning the interactions of potential targets of KBL with well-known therapeutic targets of vitiligo by using protein-protein interaction (PPI) data. As a result, key nodes with higher level of seven topological parameters, including “degree centrality (DC),” “betweenness centrality (BC),” “closeness centrality (CC),” “eigenvector centrality (EC),” “network centrality (NC),” and “local average connectivity (LAC)” were identified as the main targets in the network, followed by subsequent incorporation into the ClueGO for GO and KEGG signaling pathway enrichment analysis.

**Results:**

In accordance with the topological importance, a total of 23 potential targets of KBL on vitiligo were identified as main hubs. Additionally, enrichment analysis suggested that targets of KBL on vitiligo were mainly clustered into multiple biological processes (associated with DNA translation, lymphocyte differentiation and activation, steroid biosynthesis, autoimmune and systemic inflammatory reaction, neuron apoptosis, and vitamin deficiency) and related pathways (TNF, JAK-STAT, ILs, TLRs, prolactin, and NF-*κ*B), indicating the underlying mechanisms of KBL on vitiligo.

**Conclusion:**

In this work, we successfully illuminated the “multicompounds, multitargets” therapeutic action of KBL on vitiligo by using network pharmacology. Moreover, our present outcomes might shed light on the further clinical application of KBL on vitiligo treatment.

## 1. Background

Vitiligo, a progressive and acquired depigmentation disease, is characterized by the presence of circumscribed white macules on the skin [1]. An estimated 1–4% of the global populations are influenced by vitiligo [2], with certain subgroups at higher risk (up to 2-3%) [3]. In terms of the mechanism of pigmentation loss, a series of speculations have been proposed, including oxidative stress, autoimmunity, injured melanocyte migration, and/or proliferation as well as genetic and neural theories [4–7].

The present clinical therapeutic strategies mainly focus on the enhanced melanogenesis of melanocytes in skin lesions and the restoration of skin color to the normal level [8]. In addition, vitamin D analogs, excimer laser, and steroid therapy aim at the restoration of pigmentation [9–11], the successful rates of which, however, are restricted by the failure to follow the primary pathogenesis of vitiligo. Of note, the administration of psoralens combined with subsequent exposure to ultra-violet light (sun light) is considered as one of the most common therapies of vitiligo, nevertheless only 61% of patients could obtain over 25% repigmentation [12]. Moreover, the above therapy could induce serious adverse events, including phototoxic response, perioral dermatitis, cutaneous atrophy, and long-term carcinogenic risk [13]. To this end, accumulative efforts have been made to identify novel therapeutic medicine and to evaluate multitargets, in order to examine the complicated network of the therapeutic mechanism of vitiligo.

Traditional Chinese medicine (TCM), a comprehensive medicinal system, plays a key role in maintaining health for Asian population [14]. Accumulative prevalence has been gained on TCM in western countries due to the robust therapeutic effects and relatively few side effects. According to the theory of traditional Chinese herbal medical science, TCM is considered as promising preventive and therapeutic sources for complicated disorders, including vitiligo [15–18].

Kang-bai-ling (KBL), a Chinese patent medicine, consists of five medicinal herbs, including Psoraleae Fructus (Buguzhi, BGZ), Angelicae Dahuricae Radix (Baizhi, BZ), Stephaniae Tetrandrae Radix (Fangji, FJ), Mume Fructus (Wumei, WM), and Glycyrrhizae Radix et Rhizoma (Gancao, GC). KBL, first proposed by Professor Min Zhongsheng in 1990s, has been utilized to treat a great number of patients with vitiligo ever since. Previous studies have indicated the good curative effect of KBL in clinics [19]. The mechanism of KBL against vitiligo is related to the up-regulated expression of tyrosinase in melanin cells, enhanced activity and induced synthesis of melanin [20]. However, the scientific basis as well as potential pharmacological mechanisms of KBL is still unclear and needs further investigations.

The therapeutic effects of the majority of TCM drugs are generally regulated by diverse targets and pathways in human body due to the complicated active components of TCM, which could not easily be accurately determined through traditional approaches. Thus, it is urgent to develop novel and appropriate strategies [21]. Herein, in this study, a comprehensive approach (a combination of multiple network-based computational and algorithm-based approaches) was utilized, by combining prediction of active compounds based on multiple pharmacokinetic parameters and excavation of diverse drug targets and network analysis, aiming at illumination of the underlying mechanisms of KBL on vitiligo.

## 2. Methods

### 2.1. Data Preparation

Data for all ingredients of each herb in KBL were collected from the Chinese Academy of Sciences Chemistry Database [22] (http://www.organchem.csdb.cn/scdb/default.asp), Traditional Chinese Medicine System Pharmacology Database [23] (http://lsp.nwu.edu.cn/tcmsp.php), and relevant literatures, followed by subsequent addition into the ingredient database.

### 2.2. OB and Drug-Likeness Screening

OB prescreening, defined as the distribution degree of an oral dose of drug into bloodstream, is one of the most requisite premises in terms of oral drug discovery as well as clinical application [24]. Additionally, drug-likeness, which is defined as a qualitative concept for assessment of the structural similarity of compounds with clinical therapeutics in the DrugBank database, is determined early after drug discovery [25]. The detailed calculations of these two parameters have been reported by Wang et al. Briefly, OB = 30% along with drug-likeness index = 0.18 was defined as the cutoff value for the selection of candidate compounds [26]. In our study, several compounds, including psoralen and angelicin, were initially omitted in line with these screening criteria. Nevertheless, they were still included into candidate compounds for further analysis, due to their widely acknowledged pharmacological activities [27].

### 2.3. Potential Drug Targets Prediction for KBL

The identification of targets is considered as a pivotal stage during drug exploitation. Herein, systematic drug-targeting approach, which was proposed by Li et al. [28], was utilized for accurate determination of potential targets for medical compositions.

### 2.4. Collection of Known Vitiligo-Related Targets

Known vitiligo-associated targets were acquired from five existing resources: (1) MalaCards human disease database [29] (https://www.malacards.org); (2) OMIM database [30] (https://omim.org); (3) Therapeutic Target Database [31] (http://bidd.nus.edu.sg/group/cjttd/); (4) DrugBank database [32] (https://www.drugbank.ca); (5) Genetic Association Database [33] (https://geneticassociationdb.nih.gov). The detailed information of these known therapeutic targets is summarized in [Supplementary-material supplementary-material-1]. Finally, 98 known vitiligo-related targets were selected after redundancy deletion.

### 2.5. PPI Data

Afterwards, PPI data were retrieved from a prevalently used Cytoscape plugin BisoGenet [34] through six present PPI datasets, including Biological General Repository for Interaction Datasets (BioGRID), Database of Interacting Proteins (DIP), Biomolecular Interaction Network Database (BIND), Molecular INTeraction Database (MID), Human Protein Reference Database (HPRD), and InAct. In addition, the detailed information of these PPI datasets is summarized in [Supplementary-material supplementary-material-1].

### 2.6. Network Construction

Based on the interaction data obtained from the Cytoscape plugin BisoGenet, we first constructed an interaction network of the known vitiligo-associated targets and potential drug targets of KBL, followed by further visualization using Cytoscape (Version 3.6.0).

### 2.7. Defining of Network Topological Feature Set

We then assessed the topological property of every node in the interaction network by calculating six parameters with a Cytoscape plugin CytoNCA [35]: “degree centrality (DC),” “betweenness centrality (BC),” “closeness centrality (CC),” “eigenvector centrality (EC),” “network centrality (NC),” and “local average connectivity (LAC).” The definitions and computational formulas of these parameters have been previously defined and represent the topological importance of a node in the network. These six parameters were representatives of the topological significance of each individual node of the entire network. To be specific, a larger quantitative value indicated the greater significance of the node in this network.

### 2.8. Gene Ontology and Pathway Enrichment Analysis

GO analysis: targets were performed in Omicshare [36] for more intensive understanding of their role in three categories: biological processes (BP), molecular function (MF), and cell component (CC). A *P* value of ≤0.05 indicated statistical significance, followed by applied hypergeometric examination to identify enriched GO terms.

ClueGO [37], a widely used Cytoscape plugin to visualize the nonredundant biological terms for large clusters of genes in a functionally grouped network, was used to reflect the enrichment analysis of 23 candidate targets of KBL. The present findings were categorized into two large clusters in this study: biological processes and the signaling pathway. ClueGO network, established by kappa statistics, was used to reflect the relationships of terms in accordance with the similarity of their related genes.

## 3. Results

### 3.1. Candidate Compound Screening for KBL

Accumulative efforts have been made to clarify the therapeutic mechanisms of TCM, however, with sluggish progress on the molecular level. Due to the unavailable effective methods specifically developed for identification of the active compounds in medicinal herbs, OB screening combined with drug-likeness assessment may be a feasible strategy. In our study, a total of 123 possible compounds with proper values of these two parameters were collected from the herbal constituents of KBL. In addition, 45 compounds (such as psoralen and angelicin) with lower values of OB or drug-likeness parameter, exhibiting massive pharmacological activities, were typical components of herbal drugs (reported in literature) and were also collected as the candidate active compounds. Thus, these compounds were chosen as candidate-active compounds as well. Finally, 168 compounds of five herbs were recognized “candidate compounds” ([Supplementary-material supplementary-material-1]). To be specific, the numbers of candidate compounds in BGZ, BZ, FJ, WM, and GC was 13, 38, 9, 13, and 117, respectively. Among all the candidate compounds, 17 of them were distributed in more than one herb of KBL, with validated diverse biological activities. For instance, linoleic acid, existing in almost all the three herbs except for FJ and GC, exerts potent antioxidant, anti-inflammatory, metabolic regulation, anticancer, and immune-regulatory activities [38]. Similarly, quercetin, ursolic acid, stigmasterol, and angelicin, which were distributed in at least two herbs of KBL, also exert various pharmacological properties, participating in the regulation of multiple physiological and pathological processes, such as inflammation, immune response, and stress process [39–42].

### 3.2. Potential Target Prediction for KBL

TCM formulas were generally effective in terms of preventive and curative effects on complicated disorders, entirely dependent on the combined effects of various compounds and targets. Thus, apart from the clarification of probable active compounds of KBL, we should explore the therapeutic targets as well. Herein, potential targets of candidate compounds were predicted based on the integration of chemical, genomic, and pharmacological data. For 168 candidate compounds, 299 potential targets were predicted ([Supplementary-material supplementary-material-1]). The numbers of potential targets linked by BGZ, BZ, FJ, WM, and GC were 86, 96, 71, 232, and 275, respectively. Despite the different numbers of targets related to each herb, significant overlaps were observed in the five herbs, indicating that diverse components in KBL may exert congeneric effects possibly by modulating the similar targets. For instance, both BGZ and BZ have been validated to affect the pathogenesis and progression of vitiligo through regulating immune- and inflammation-related pathways and promoting both adhesion and migration of melanocytes [43].

**Figure 1:**
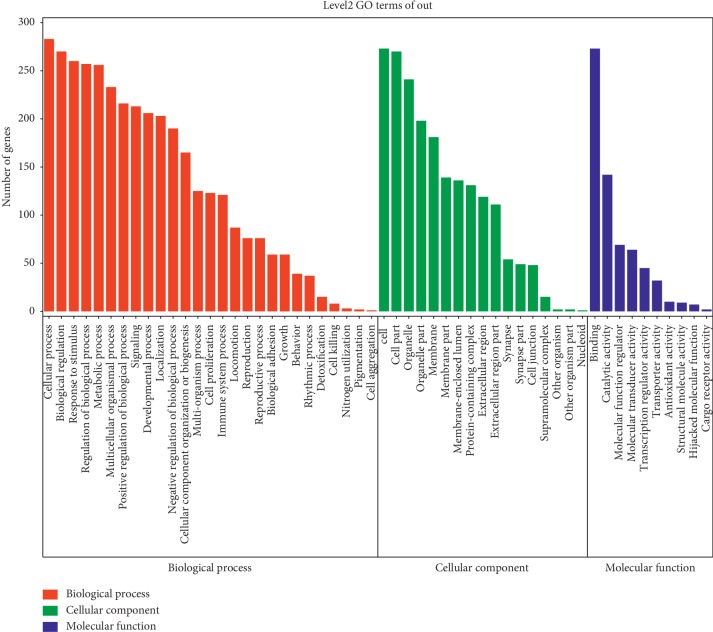
Gene ontology analysis of the potential targets of KBL. Potential targets of KBL was performed in Omicshare to gain more insights into their involvement in various Biological Processes (red section), Molecular Function (blue section) and Cell Component (green section). We considered a *P* value cutoff of ≤0.05 as significant and applied hypergeometric test to identify enriched GO terms. Terms of same category are ordered by *P* values, left terms are more significant. Information of the number of involved genes in a term are shown in *y*-axis.

For further understanding of the roles of the potential targets involved in diverse biological processes, cellular component as well as molecular functions, preliminary GO (Gene Ontology) analysis was conducted with Omicshare, a free database based on DAVID, revealing that the potential targets were enriched in response to various types of stimuli (ROS, chemical, drugs, inflammatory factors and hormone), immune systems, cellular metabolism, cell-cell communication, cell growth, and death and development, all of which participated in the pathologic process of vitiligo (Figure 1 and [Supplementary-material supplementary-material-1]) [44–47].

**Figure 2:**
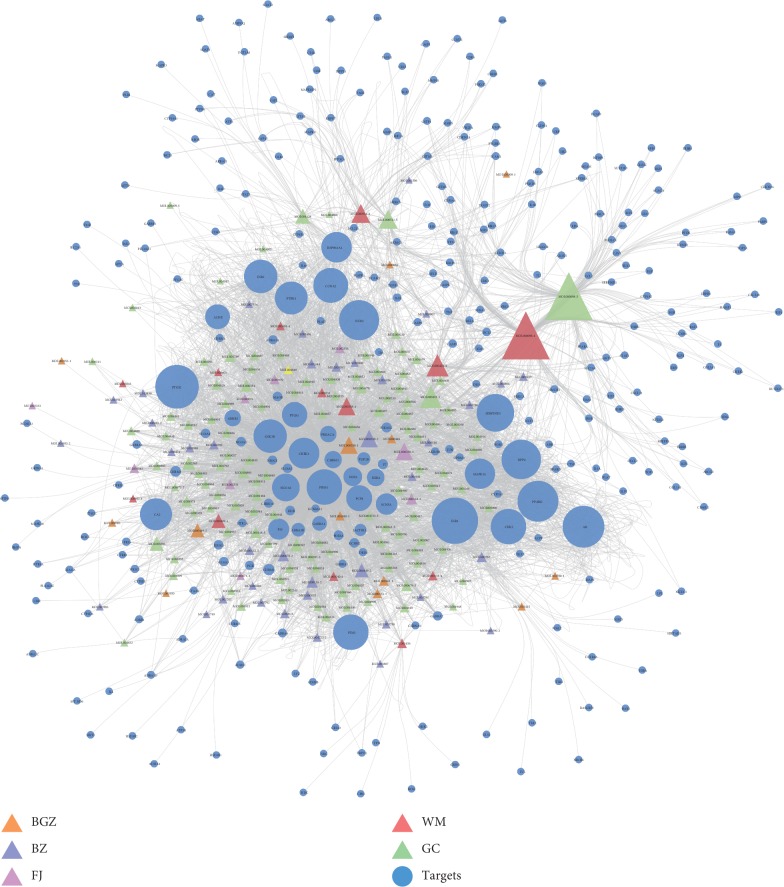
Construction of the KBL compound-potential target network. The compound-potential target network was constructed by linking the candidate compounds and their potential targets of the 5 herbs, which are constituents of KBL. The nodes representing candidate compounds are shown as polychrome triangle and the targets are indicated by blue circular.

For more comprehensive understanding of KBL component-targets network from a systematic and holistic perspective, Cytoscape was used to construct a network map, which altogether consisted of 490 nodes as well as 4303 edges (Figure 2). To be specific, the degree of node indicated the number of edges or targets correlating with node based on the topological analysis. In our network, 93 candidate components were identified with a median ≥20 degrees. To be specific, quercetin, kaempferol, and ursolic acid acted on 162, 72, and 61 targets, respectively, which were therefore considered as the critical bioactive ingredients in KBL. Quercetin has been demonstrated to decrease hydrogen peroxide-induced intracellular redox imbalance in melanocyte and thereby achieving repigmentation by regulating certain crucial pathways [48, 49]. In addition, kaempferol and ursolic acid have also been validated to increase melanin content via positively regulating the activity of tyrosinase directly [50]. These findings might shed novel light on the pleiotropic effects of TCM herbs.

### 3.3. Collection of Known Vitiligo-Related Targets

**Figure 3:**
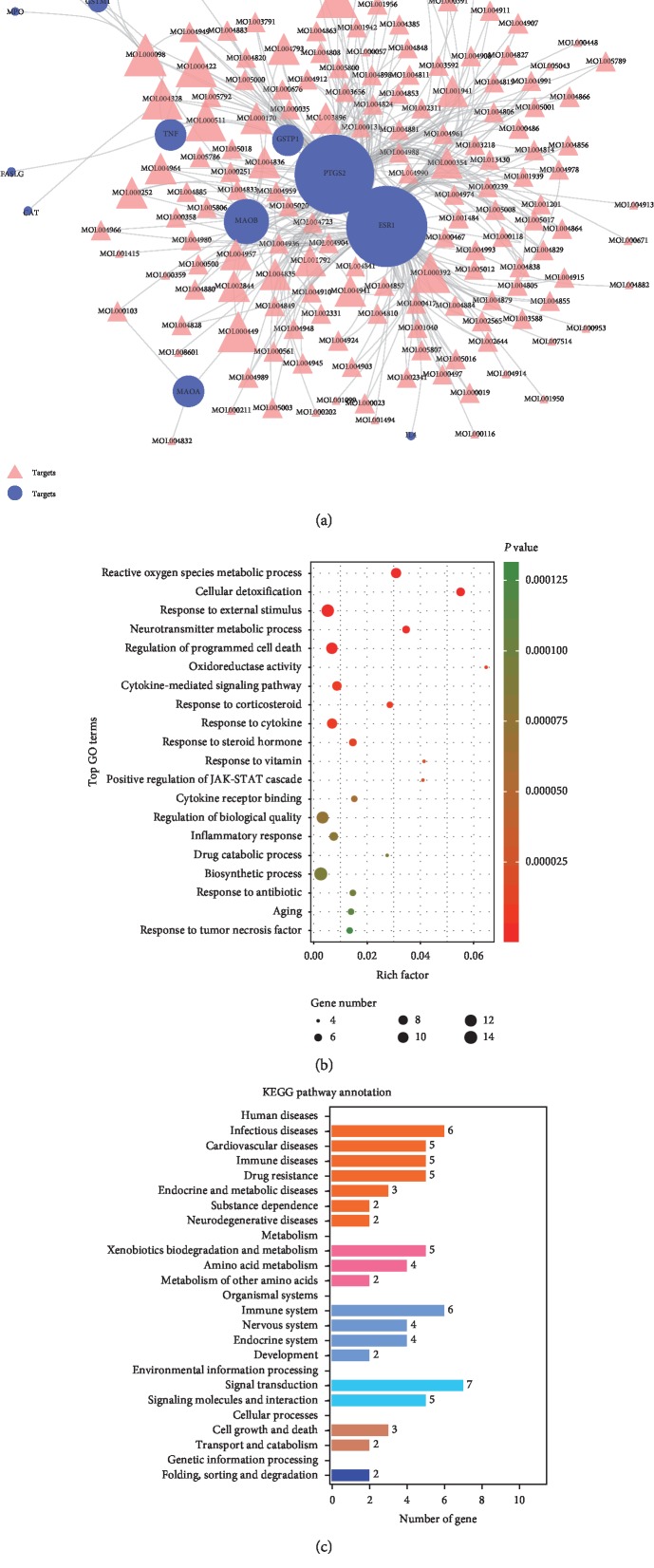
KBL shared 15 potential targets with known pathological course related targets of vitiligo. (a) The compound potential target network was constructed by linking the overlapped targets (between KBL potential and known vitiligo-related) and the homologous candidate compounds of KBL. The nodes representing candidate compounds are shown as pink triangle and the targets are presented as purple circular. (b) 15 overlapped targets (between KBL potential and known vitiligo-related) was performed in Omicshare to gain more insights into their involvement in various GO terms. We considered a *P* value cutoff of ≤0.05 as significant and applied hypergeometric test to identify enriched GO terms. Following chart shows an overview of the gene ontology analysis with up to 20 significantly enriched terms. (c) 15 overlapped targets (between KBL potential and known vitiligo-related) was performed in Omicshare to gain more insights into their involvement in KEGG pathways.

Vitiligo is a polygenic predisposing disorder. Genetic-environmental interaction has been revealed to play a role in the pathogenesis of vitiligo. Herein, 98 vitiligo-related targets were retrieved from five present resources. Notably, 15 of the identified potential targets of the KBL were also the well-recognized vitiligo disease (or therapeutic drugs)-related targets, indicating the potential therapeutics of this formula (Figure 3(a) and [Supplementary-material supplementary-material-1]).

To understand the possible pharmacological mechanisms of KBL on vitiligo, GO and KEGG enrichment analyses were performed on the above-described 15 targets (Figures 3(b) and 3(c) and Tables [Supplementary-material supplementary-material-1] and [Supplementary-material supplementary-material-1]). Consistent with the current pathological mechanism of vitiligo, they were mainly enriched in homeostatic process, cell growth and death, cellular metabolism, immune and inflammatory responses, and neuroendocrine signal conduction. Moreover, KBL was found to modulate vitiligo via the regulation of activity of multiple series enzymes such as tyrosinase (TYR), oxidoreductase (GSTP1, CAT, MPO, and GSTM1), and controlling signal transducer (NFE2L2). Furthermore, inflammatory factors and interleukin (IL) signaling pathway (IL4 and IL10), adaptive immune response (IFNG), and cell apoptosis (FASLG and TNF) also played critical roles in the clarification of the mechanisms of KBL on vitiligo. Notably, vitiligo has been accumulatively reported to suffer from uncontrolled neurocrine regulation, all of which affect every situation and bring greater challenges to vitiligo treatment [51]. In the present study, our analysis suggested that KBL might be of therapeutic effects on vitiligo by affecting catecholamine metabolism, neurotransmitter degradation (MAOA and MAOB), and intracellular steroid hormone receptor signaling pathway (ESR1). Consistent with previous outcomes, we confirmed that KBL exerted a curative role on not only melanocytes but also neuroendocrine system from the network pharmacologic level. Vitiligo-related drugs causing systemic alterations would not only control vitiligo but might play also a therapeutic or side role on other disorders. By contrast, drugs regulating systemic alterations could affect vitiligo as well. Taken together, we hypothesized that vitiligo might respond towards systemic neuro-immuno-inflammatory disorder in the skin tissue, which could be systematically controlled by KBL.

### 3.4. Identification of Candidate Targets for KBL against Vitiligo

As indicated by recent evidences in network biology, disease-related genes or proteins do not work alone; instead, there are diverse interactive pathways and molecular networks on various levels. To clarify the pharmacological mechanism by which KBL ameliorates vitiligo, we constructed protein-protein networks that may reflect the behavior and properties of biological molecules. To this end, a potential target network (394 nodes and 24238 edges) and a defined vitiligo-associated target network (295 nodes and 14043 edges) were established by utilizing PPI data of these genes, respectively. We then merged these two networks to obtain a core protein-protein interaction (CPPI) network that consisted of 87 nodes and 1946 edges. Subsequently, candidate antivitiligo targets of KBL were screened by using the topological features of CPPI. A node with the topological features exceeding that of median of all nodes was considered as a hub in the network (candidate targets in our study). As a result, the candidate targets were identified according to a widely used plugin CytoNCA, six topological features, including “DC,” “BC,” “CC,” “EC,” “NC,” and “LAC.” The median values of “DC,” “BC,” “CC,” “EC,” “NC,” and “LAC” were 43, 23.3953542332676, 0.666666666666666, 0.103930160403251, 37.2721127301037, and 35.15, respectively. Therefore, 23 candidate targets, with “DC” >43, “BC” >23.3953542332676, “CC” >0.666666666666666, “EC” >0.103930160403251, “NC” >37.2721127301037, and “LAC” >35.15, were identified. The detailed topological characteristics and the PPI network are shown in [Supplementary-material supplementary-material-1].

### 3.5. Enrichment Analysis of Candidate Targets for KBL against Vitiligo

**Figure 4:**
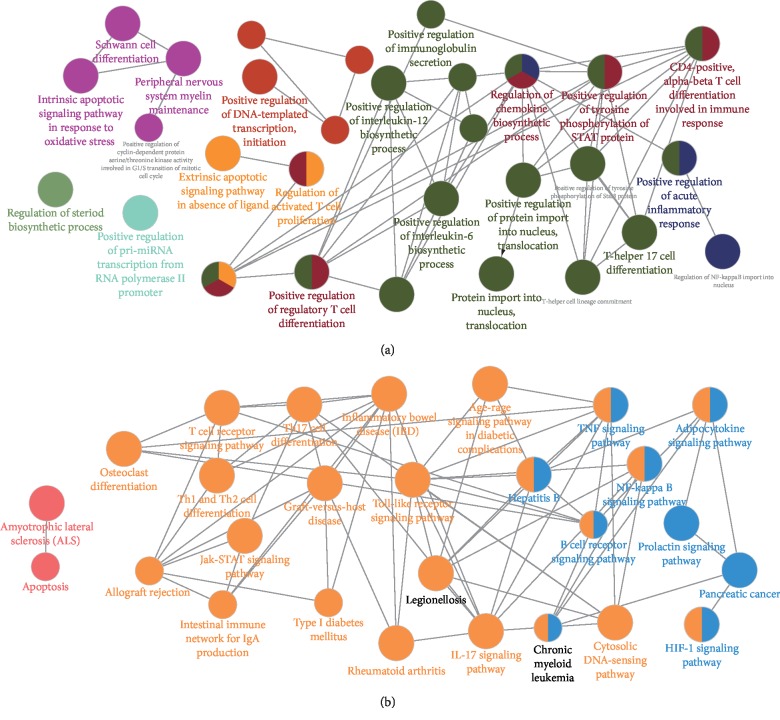
Enrichment analysis of candidate targets for KBL against vitiligo. The enrichment analysis is generated by ClueGo and the most vital term in the group is labeled. Functionally related groups partially overlap. Representative enriched biological process or pathway (*P* < 0.05) interactions among main KBL targets. (a) Candidate KBL targets enriched in the representative biological process are shown. (b) Candidate KBL targets enriched in the representative signaling pathway are shown.

To further reveal the probable role of 23 candidate targets, ClueGO, an extensively utilized Cytoscape plugin, was employed to find out interrelations of functional groups with their potential scientific connotation in biological networks, followed by division into GO biological processes as well as signaling pathway (Figure 4). To be specific, biological processes were mainly associated with DNA translation, lymphocyte differentiation and activation, STAT signal transduction, steriod biosynthesis, and peripheral nervous system myelin maintenance (Figure 4(a)). And multiple pathological processes and diseasees, including inflammatory cytokine stimulation, intracellular ROS accumulation, neuron apoptotic process, and vitamin deficiency, could indirectly affect the progression of vitiligo, providing potent evidence for our speculation that KBL exerted a curative role on vitiligo via systemic neuro-immuno-inflammatory modulation from a genetic perspective.

The involved pathway was mainly composed of TNF, JAK-STAT, ILs, TLRs, NF-*κ*B, prolactin, lymphocyte differentiation-related, and autoimmune and systemic inflammatory reaction-related pathways (Figure 4(b)). The above pathways could further be categorized into three representative modules, namely, immunoregulation, melanogenesis, and neuroendocrine regulation. Inflammation and immune mechanisms are prone to play a role in the melanocyte destruction of vitiligo, while ILs, TLRs, and JAK-STAT signaling pathways participate in regulating proinflammatory and immune reactions, as well as lymphocyte differentiation. In addition, destructive melanocytes are well-known characteristics of vitiligo. NF-*κ*B, which is capable of decreasing apoptosis of vitiliginous keratinocytes, thereby makes contributions to the proliferation as well as differentiation of melanocytes, further promoting melanin biosynthesis. Moreover, there is intimate interaction between melanocytes and nerve fibers as well as extracellular matrix, while mediators might directly modulate the functions of melanocytes. Hyperactive prolactin pathway is likely to cause hyperproduction of H_2_O_2_, which is capable of not only changing calcium homeostasis to influencing vitiligo progression via the modulation of synaptic transmission, neuronal excitability, and neurosecretion but also inducing the direct death of melanocytes. To sum up, the present outcomes demonstrate the vital pharmacological mechanism of KBL against vitiligo and also shed novel light on the drug exploitation strategy of vitiligo.

## 4. Discussion

Vitiligo, the most prevalent depigmentation skin disease, is caused by dysfunction or destruction of melanocytes, the major pigment-producing cells [52]. Vitiligo is psychologically destructive in general, though it is not physically harmful [53]. The present accessible therapeutic regimens, such as steroid therapy, vitamin D analogs, and excimer laser, are not proper for the majority of patients due to the complicated, ineffective, and time-consuming effects [54, 55]. Large-scale GWAS, mainly performed in European-derived white and Chinese populations, have reported about 56 different genetic loci contributed to vitiligo risk, part of which also made contribution to oxidative stress, melanogenesis of melanocytes, neuroendocrine modulation, and autoimmunity [56, 57]. Thus, better understandings of the possible mechanisms might contribute to more specificity and effectiveness of the therapeutic regimens.

The 30-year application KBL has proved its practical effectiveness on vitiligo, however, without systematical understanding of the pharmacological mechanisms. In our study, network pharmacology was employed to further investigate the mechanisms of KBL on vitiligo. To this end, we mainly focused on three aspects. Firstly, common targets of KBL of vitiligo were enriched in pathological changes of intracellular redox equilibrium and ion homeostasis. Secondly, enrichment analysis showed the regulatory effects of KBL on the differentiation of T-lymphocytes and that the cytokines produced may be the most important mechanism of vitiligo treatment. Thirdly, neuroendocrine regulation was also considered as the critical sections of KBL on vitiligo therapy. To sum up, the therapeutic effect of KBL on vitiligo was mediated by controlling the levels of its targets, which have been shown to be critically involved in vitiligo progression in a multicomponent, multitarget, and multilink pattern.

As is known to all, lymphocytes, especially T-lymphocytes along with T cells-generated cytokines, play crucial roles in vitiligo progression, as indicated by multiple research studies. Due to the advanced inflammation speculations, the imbalance of Th1/Th2 has become a paradigm in vitiligo pathogenesis, which can affect on a variety of inflammatory cytokines, such as ILs [58, 59]. The latest studies have shown that various immune and inflammatory cells can synthesize and secrete neurotrophic factors via the enhancement of Th2 cells, induction of mast cell proliferation and differentiation, activation of eosinophils, intensification of immune response, and promotion of the secretion of inflammatory mediators. Similarly, we have also demonstrated that there are diverse critical signaling pathways related to T-lymphocytes differentiation (TLRs) [60] regulated by KBL on vitiligo therapy. Furthermore, studies have shown that nervous pathways affect the release of neuropeptides (NPs) and various cell behaviors, subsequently leading to changes on costimulatory molecules and cytokine expression of innate and adaptive immunity in the skin, thereby affecting the pathogenesis and progression of vitiligo [51, 61]. Collectively, we cautiously speculate that vitiligo might be characterized by systemic neuro-immuno-inflammatory response, which is consistent with the holistic understanding of both physiological and pathological states of the human body in TCM concept.

Despite the valuable discoveries, there are still certain limitations. To begin with, some compounds of herbs in KBL were neglected in consideration of the inadequate data obtained from existing databases and laboratory findings. Secondly, there was a lack of animal assay or clinical trial for validation of our speculations. Thirdly, there is great difficulty in the direct evaluation of specific targets and critical active ingredients, which are retrieved from a chemically complicated as well as cognitively rare TCM formula. Therefore, future studies are warranted to confirm these potential mechanisms of KBL on vitiligo from animal assays and clinical trials. In addition, we aim to intensively explore (1) the upstream and downstream mechanisms of this formula in regulating these pathways and (2) the difference in the material basis in regulating these different pathways.

## Data Availability

The data used to support the findings of this study are available from the corresponding author upon request.

## Supplementary Materials

Supplementary Materials Table S1: all the candidate compounds of KBL. Table S2: all the potential targets of KBL. Table S3: known vitiligo-related targets. Table S4: KBL shared 15 potential targets with known vitiligo-related targets. Table S5: topological feature values of all/significant/candidate targets for KBL against vitiligo Table S6: detailed information on six existing protein-protein interaction databases. Table S7: gene ontology analysis of the potential targets of KBL. Table S8: GO enrichment analysis of the 15 overlapped (between KBL potential and known vitiligo-related) targets. Table S9: KEGG enrichment analysis of the 15 overlapped (between KBL potential and known vitiligo-related) targets.
